# Comparison of antibody and T cell responses elicited by BBIBP-CorV (Sinopharm) and BNT162b2 (Pfizer-BioNTech) vaccines against SARS-CoV-2 in healthy adult humans

**DOI:** 10.1007/s11357-021-00471-6

**Published:** 2021-10-11

**Authors:** István Vályi-Nagy, Zsolt Matula, Márton Gönczi, Szabolcs Tasnády, Gabriella Bekő, Marienn Réti, Éva Ajzner, Ferenc Uher

**Affiliations:** 1 Department of Hematology and Stem Cell Transplantation, Central Hospital of Southern Pest, National Institute of Hematology and Infectious Diseases, Budapest, 1097 Hungary; 2 Laboratory for Experimental Cell Therapy, Central Hospital of Southern Pest, National Institute of Hematology and Infectious Diseases, Nagyvarad ter 1., Budapest, H-1097 Hungary; 3 Central Laboratory of Central Hospital of Southern Pest, National Institute of Hematology and Infectious Diseases, Budapest, 1097 Hungary

**Keywords:** Adaptive immunity, Anti-SARS-CoV-2 antibodies, IFNγ-producing T cells, IFNγ ELISpot assay, Vaccination

## Abstract

In the present study, humoral and T cell-mediated immune responses elicited by BBIBP-CorV (inactivated virus) and BNT162b2 (mRNA-based) vaccines against SARS-CoV-2 virus were compared. Convalescent volunteers were also investigated to evaluate adaptive immunity induced by live virus. Although both vaccines induced antibody- and T cell-mediated immune responses, our analysis revealed significant quantitative and qualitative differences between the two types of challenges. The BBIBP-CorV vaccine elicited antireceptor-binding domain (RBD) IgG, as well as anti-spike protein (S) IgG and IgA antibodies in healthy individuals, the levels of which were much lower than after BNT162b2 vaccination but still higher than in the convalescent patients. The cumulative IFNγ-positive T cell response, however, was only twofold higher in participants injected with BNT162b2 compared to those who were primed and boosted with BBIBP-CorV vaccine. Moreover, the inactivated virus vaccine induced T cell response that targets not only the S but also the nucleocapsid (N) and membrane (M) proteins, whereas the mRNA vaccine was able to elicit a much narrower response that targets the S protein epitopes only. Thus, the pattern of BBIBP-CorV-induced T cell response in virus-naive participants was similar to the cell-mediated anti-SARS-CoV-2 response observed in convalescent patients. Based on these data, we can conclude that the BBIBP-CorV inactivated virus vaccine is immunologically effective. However, the duration of BBIBP-CorV-induced integrated, antibody, and T cell-mediated, immune responses needs further investigation.

**Supplementary Information:**

The online version contains supplementary material available at 10.1007/s11357-021-00471-6.

## Introduction

Severe acute respiratory syndrome coronavirus-2 (SARS-CoV-2), the causative agent of coronavirus disease 2019 (COVID-19), emerged in 2019, continues to cause significant morbidity and mortality worldwide [1]. Thus, it is clear that effective vaccines represent the only viable road to get out of the pandemic. Fortunately, several vaccines and vaccine candidates were developed or are under development, including nucleic acid, adenovirus-vectored, inactivated virus, and subunit protein vaccines [2, 3].

Currently, one vaccine against SARS-CoV-2 that involves inactivated virus technology has been approved for emergency use in Hungary (BBIBP-CorV, Sinopharm). Unfortunately, however, data mainly about antibody responses elicited by BBIBP-CorV have been reported to date, whereas the existence and magnitude of the vaccine-induced T cell response are less clear [4–6]. To address this knowledge gap, we compared antibody levels and T cell responses in 57 adult healthy volunteers who received either the BBIBP-CorV or the more thoroughly characterized BNT162b2 (Pfizer-BioNTech) [7–9] vaccine. Specimens from 35 convalescent individuals were also investigated as additional control. We used serological assays and flow cytometry, as well as T cell ELISpot technology to detect IFN-γ release from immune cells after exposure to SARS-CoV-2 spike (S1 and S2), nucleocapsid (N), membrane (M), and envelop (E) peptides.

We found that the BBIBP-CorV vaccine induces reasonable anti-RBD and anti-S1/S2 IgG as well as anti-S1, anti-S2, anti-N, and anti-M T cell responses following the second dose.

## Materials and methods

### Study participants and design

**Fig. 1:**
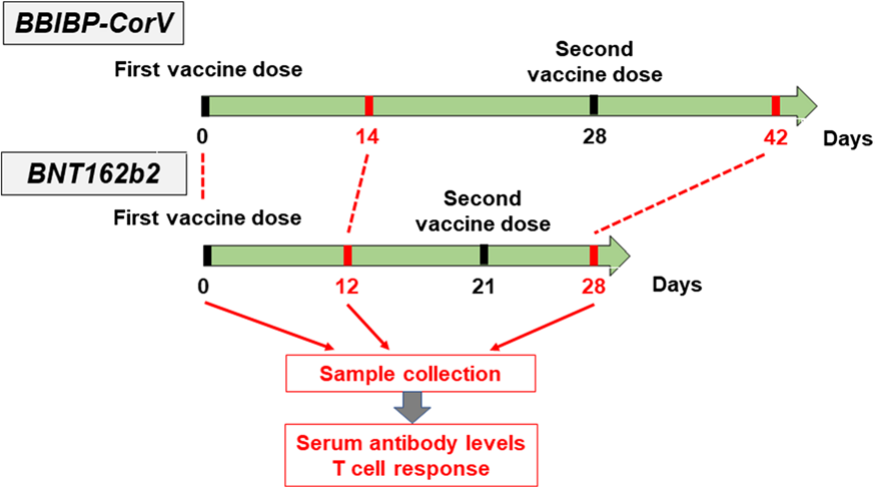
Vaccination schedule and sample collection. Study participants received a priming and a booster vaccine dose with BBIBP-CorV (on days 0 and 28) or BNT162b2 (on days 0 and 21) following the manufacturer’s guidance and instructions. Blood samples were obtained on days 0 (prior vaccination), 12 or 14 (post-prime), and 28 or 42 (post-boost)

We assessed longitudinal SARS-CoV-2-specific antibody and T cell responses pre- and post-vaccination among 57 healthy adult volunteers, out of whom 25 received the BBIBP-CorV (Sinopharm’s Beijing Institute of Biological Products, Beijing, China) and 32 the BNT162b2 (Pfizer-BioNTech, Pfizer Inc., NY, USA) vaccine with no known history of COVID-19 (Table 1). Additional exclusion criteria were fever, cough, and diarrhea 1 week before vaccination. Specimens were obtained between 01.13.2021 and 05.11.2021 at three time points: prior vaccination, 12–14 days after the first vaccine dose, and 8–14 days after the second dose (see Fig. 1). Specimens from polymerase chain reaction (PCR) confirmed COVID-19 (with mild or moderate disease) convalescent individuals (*n* = 35) were used as positive control. Non-responder (seronegative) convalescent volunteers were excluded from this study.

**Table 1:** Baseline characteristics of participants in this study

	BBIBP-CorV cohort		BNT162b2 cohort		Convalescent individuals (*n* = 35)
	Group 1 (*n* = 20)	Group 2 (*n* = 5)	Group 1 (*n* = 27)	Group 2 (*n* = 5)	
IFN-γ^+^ anti-SARS-CoV-2 T cells per 250,000 PBMC before vaccination (SFU)	˂ 40	≥ 40	˂ 40	≥ 40	─
Age (median; age range)	43.8 (19–63)	41.0 (35–52)	43.6 (22–65)	35.4 (25–60)	36.0 (21–66)
Sex					
Female	15	2	10	4	16
Male	5	3	17	1	19

This study was approved by the Institutional Review Board of the Central Hospital of Southern Pest – National Institute of Hematology and Infectious Diseases. Written informed consent was obtained from all participants before enrollment.

### Assessments

Venous blood was collected in vacutainer tubes, and serum samples were stored at − 80 °C. Anti-SARS-CoV-2 antibodies were detected using commercially available test systems: SARS-CoV-2 Surrogate Virus Neutralization Test (sVNT) Kit (GenScript Biotech B. V., Leiden, Netherlands) measuring IgG levels against SARS-CoV-2 RBD, LIAISON® SARS-CoV-2 S1/S2 IgG test (DIASORIN S.P.A., Saluggia, Italy) detecting anti-S1/S2 IgG antibodies, SARS-2 Covid N IgG Architect (Abbott Laboratories, Abbott Park, IL, USA) detecting SARS-CoV-2 nucleocapsid-specific IgG, and SARS-2 Covid S IgA (EUROIMMUN Medizinische Labordiagnostika AG, Lübeck, Germany) measuring IgA levels against SARS-CoV-2 spike protein.

To measure the lymphocyte subpopulations, the 6-color TBNK kit (BD Biosciences, San Jose, USA) was used, according to the manufacturer’s guide. The samples were EDTA coagulated whole blood samples. The kit uses a lyse-no-wash staining procedure, and to give absolute cell numbers, we used the single platform method with BD Trucount tubes (included in the kit).

To evaluate SARS-CoV-2-specific T cell immunity in vaccinated and convalescent individuals, freshly isolated peripheral blood mononuclear cells (PBMC) were isolated by density gradient centrifugation using the Leucosep Kit (Oxford Immunotec Ltd, Abingdon, Oxfordshire, UK). Altogether 250,000 recovered PBMCs were plated into each well of a T-SPOT® Discovery SARS-CoV-2 (Oxford Immunotec) kit that quantifies IFNγ-producing T cells in response to viral peptides. The kit is composed of five different but overlapping peptide pools to cover protein sequences of five different SARS-CoV-2 antigens including S1, S2, N, M, and E. Peptides that showed high sequence homology to endemic coronaviruses were removed from the peptide pools by the manufacturer. The cumulative spot-forming units (SFU) per 2.5 × 10^5^ PBMC of individuals were calculated as the sum of T-SPOTs for S1, S2, N, M, and E antigens minus the background.

### Statistical analysis

All statistical analyses were performed applying two-tailed unpaired *t* test or Wilcoxon signed rank test as appropriate. *p* < 0.05 was considered to be statistically significant (*p < 0.05; **p < 0.01; ***p < 0.001). Both the Wilcoxon test and descriptive statistical analysis such as median and range were calculated using non-transformed data.

### Data availability

All data, materials and methods used in the analysis will be available from the corresponding author by request, for purposes of reproducing or extending the analysis.

## Results

Blood specimens were obtained prior to vaccination (day 0), 12–14 days following the first dose, and 7–14 days following the second dose as shown in Fig. 1. Although five individuals in the BBIBP-CorV vaccinated cohort and five in the BNT162b2 vaccinated cohort already tested positive in the anti-SARS-CoV-2 ELISpot assay (i.e., participants had ≥ 40 SFU/2.5 × 10^5^ PBMC) at day 0 and some but not all of them were seropositive as well, two groups were formed among cohorts. Seronegative participants with ˂ 40 SFU/2.5 × 10^5^ PBMC were considered as virus-naive individuals (first groups), while participants with ≥ 40 SFU/2.5 × 10^5^ PBMC were considered as individuals probably experienced asymptomatic or mild SARS-CoV-2 infection earlier (second groups) (Table 1). (All participants tested negative for active infection at day 0 by PCR assay.) In unimmunized, healthy volunteers, the number of spot-forming cells was always very low (median 13.2, range 1–25, *n* = 10).

### Humoral immune response to vaccination

**Fig. 2:**
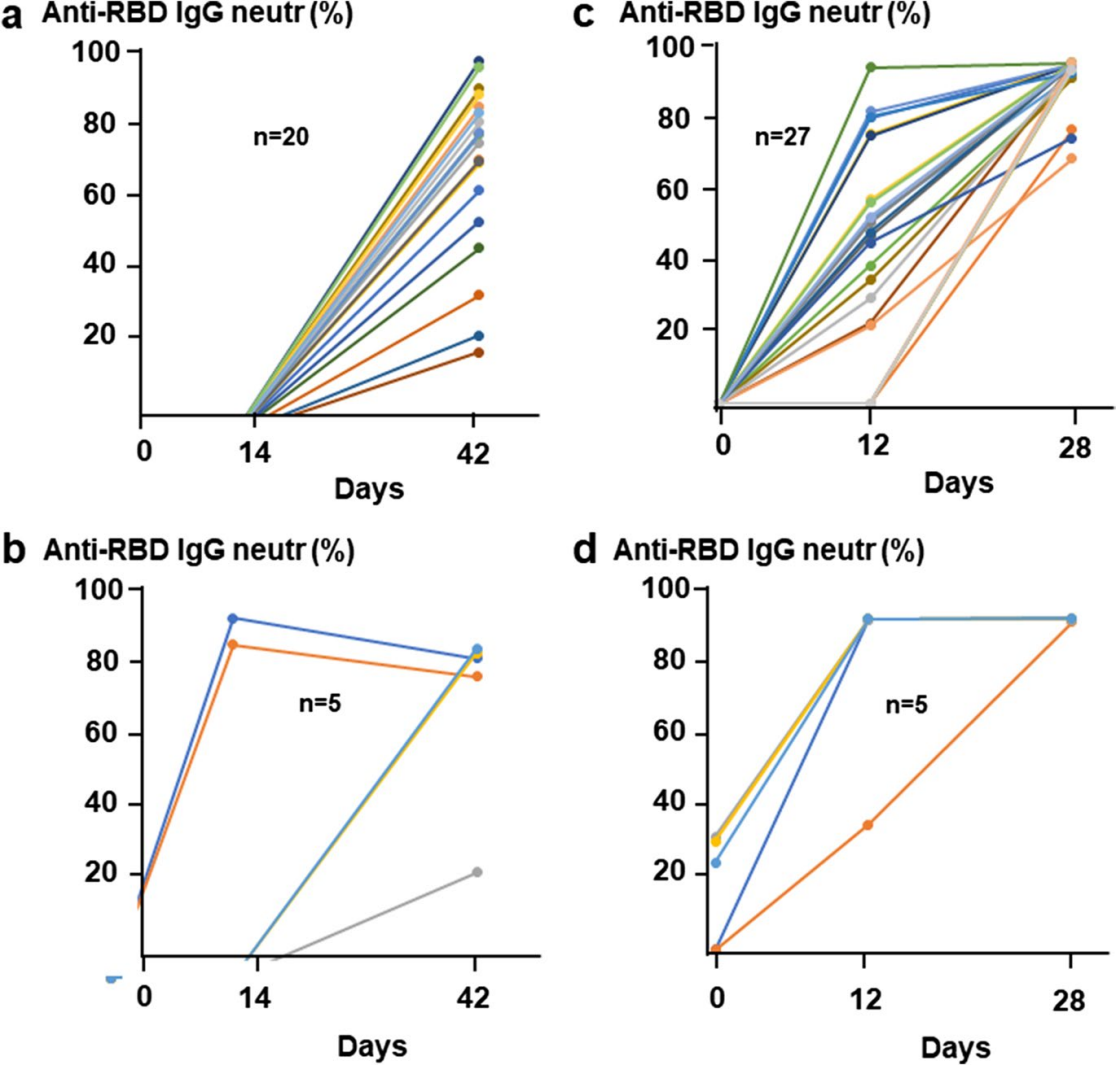
Serum anti-RBD IgG levels over time in response to different SARS-CoV-2 vaccines. Vaccination schedule and serum sampling are described in Fig. 1. The relative amounts of serum anti-RBD (“neutralizing”) IgG for 25 participants that received the BBIBP-CorV vaccine (**a** and **b**) and for the 32 participants that received the BNT162b2 vaccine (**c** and **d**) were measured by the SARS-CoV-2 surrogate virus neutralization test (sVNT). Different panels demonstrate antibody levels in individuals with (**b** and **d**) or without (**a** and **c**) preexisting T cell immunity (≥ 40 or ˂ 40 SFU/2.5 × 105 PBMC, respectively) at day 0. Connected lines indicate repeated measurements from the same subjects

While all participants in both cohorts produced specific IgG antibodies against SARS-CoV-2 RBD (surrogate virus neutralization test), the levels were significantly lower in BBIBP-CorV vaccinated naive participants compared to the BNT162b2 naive group (medians 71.0% versus 99.4%, *p* ˂0.01) after the second dose. Especially, BBIBP-CorV was unable to elicit any detectable anti-RBD IgG after the first vaccination (Fig. 2a, c). In the case of the SARS-CoV-2 experienced groups, however, two out of five BBIBP-CorV and all BNT162b2 vaccinated volunteers were already strong seropositive after the first vaccination (Fig. 2b, d).

**Fig. 3:**
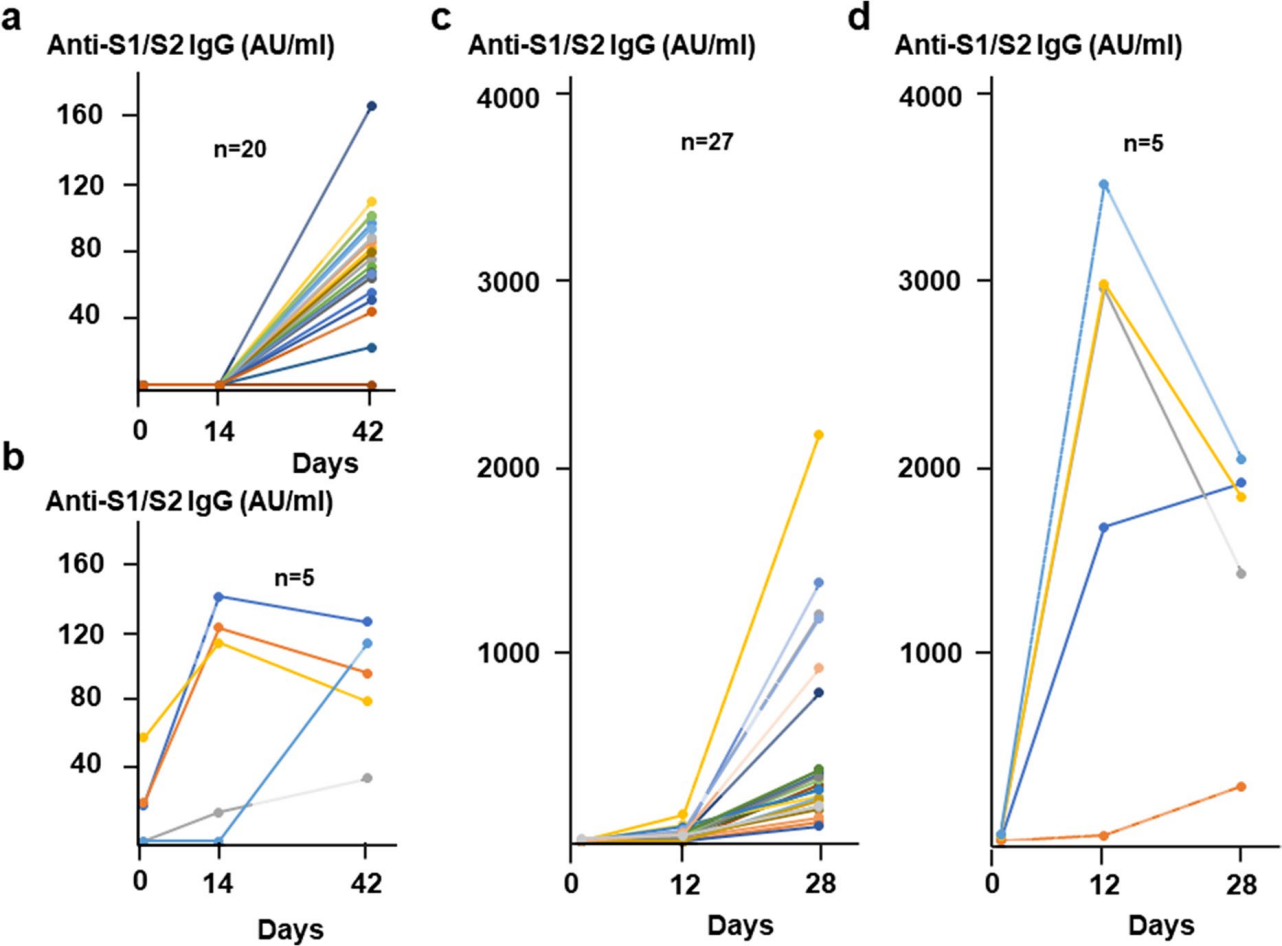
Serum anti-S1/S2 IgG levels over time in response to different SARS-CoV-2 vaccines. Vaccination schedule and serum sampling are described in Fig. 1. The amounts of serum anti-S1/S2 IgG for 25 participants that received the BBIBP-CorV vaccine (**a** and **b**) and for the 32 participants that received the BNT162b2 vaccine (**c** and **d**) were measured by the LIAISON® SARS-CoV-2 S1/S2 IgG test. Different panels demonstrate antibody levels in individuals with (**b** and **d**) or without (**a** and **c**) preexisting T cell immunity (≥ 40 or ˂ 40 SFU/2.5 × 105 PBMC, respectively) at day 0. Connected lines indicate repeated measurements from the same subjects

Consistent with these observations, anti-S1/S2 IgG levels were 6–sevenfold lower in BBIBP-CorV vaccinated naive individuals (median 80.5 AU/ml) compared with the BNT162b2 naive group (median 517.8 AU/ml, *p* ˂ 0.001) after boosting (Fig. 3a, c). The difference was even much higher, ~ 15-fold (medians 105.2 versus 1523.2 AU/ml, *p* ˂0.001) in the virus-experienced groups (Fig. 3b, d). One person from the first group of BBIBP-CorV cohort stayed seronegative even after the second dose. It has to be noted that the S1/S2-specific IgG levels were ~ 13-fold lower in the sera of convalescent patients compared with the mRNA-vaccinated naive participants (medians 41.1 versus 517.8 AU/ml, *p* ˂ 0.001).

For BBIBP-CorV vaccinees, 7 of the 20 individuals in the virus-naive group and 4 of the 5 participants in the virus-experienced group had anti-nucleocapsid protein IgG antibodies after boosting. In contrast, only one BNT162b2-injected subject was anti-N IgG positive after the second dose (Supplementary Table 1).

Spike antigen-specific serum IgA was detected throughout the entire BNT162b2 vaccinated cohort, whereas only 14 of the 20 virus-naive BBIBP-CorV vaccinees were positive after the second dose (data not shown and Supplementary Fig. 2a, b).

### Phenotype of blood lymphocytes

For analyses of main lymphocyte subsets in BBIBP-CorV vaccinated individuals, we defined the absolute numbers of CD45^+^ lymphoid cells, CD4^+^ and CD8^+^ T lymphocytes, and B and NK cells in their blood samples by flow cytometry. As shown in Supplementary Fig. 1, the first vaccination dose induced a characteristic but not statistically significant (probably because of the small sample size) expansion of CD3^+^, CD3^+^CD4^+^, and CD3^+^CD8^+^ T cell subsets, which was not further boosted with the second dose. In terms of numbers, these increases were 203.72, 131.51, and 61.41 cells per microliter blood for the CD3^+^, CD3^+^CD4^+^, and CD3^+^CD8^+^ lymphocytes in virus-naive participants at day 14, respectively (Supplementary Fig. 1a, c, e).

**Fig. 4:**
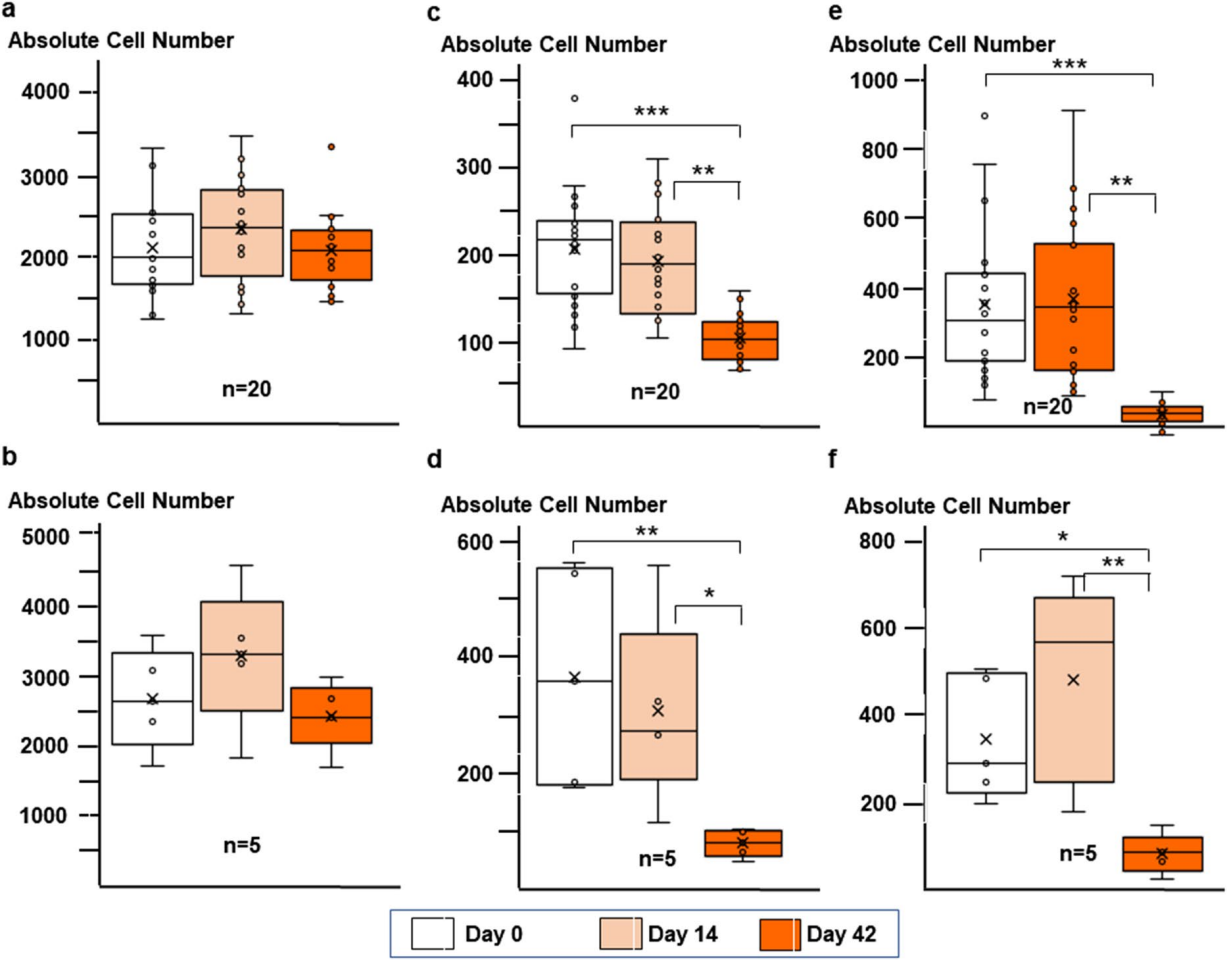
Lymphocyte landscape in participants vaccinated with BBIBP-CorV. Box plots of major lymphocyte subsets from PBMCs of individuals immunized with BBIBP-CorV. Different panels demonstrate the absolute numbers of CD45^+^ cells (**a** and **b**), CD19^+^ B cells (**c** and **d**), and CD3^−^CD16^+^CD56^+^ NK cells (**e** and **f**) in individuals with (**b**, **d**, and **f**) or without (**a**, **c**, and **e**) preexisting T cell immunity at day 0. Box plots display the median values with the interquartile range (lower and upper hinge) and ± 1.5 fold the interquartile range from the first and third quartile (lower and upper whiskers). *p* values are determined by the Wilcoxon signed rank test, and *p* < 0.05 was considered to be statistically significant (*p < 0.05; **p < 0.01; ***p < 0.001)

In contrast, for the CD19^+^ B and CD3^−^CD16^+^CD56^+^ NK cells, a marked, statistically significant reduction of absolute cell numbers was observed in both BBIBP-CorV vaccinated groups after boosting (days 42) (Fig. 4). This decrease of mature B cell numbers in the circulation is consistent with the increased anti-RBD and anti-S1/S2 IgG serum levels after boosting (see above, Figs. 2a and b and 3a and b), indicating the differentiation of CD19^+^ B cells into antibody-secreting CD19^−^ plasma cells. NK cells could also be activated and, similarly to mature plasma cells, migrated into the bone marrow and lymph nodes after the second vaccine dose. The phenotype of blood lymphocytes in the BNT162b2 cohort was not examined in this setting.

### T cell immunity following vaccination

**Fig. 5:**
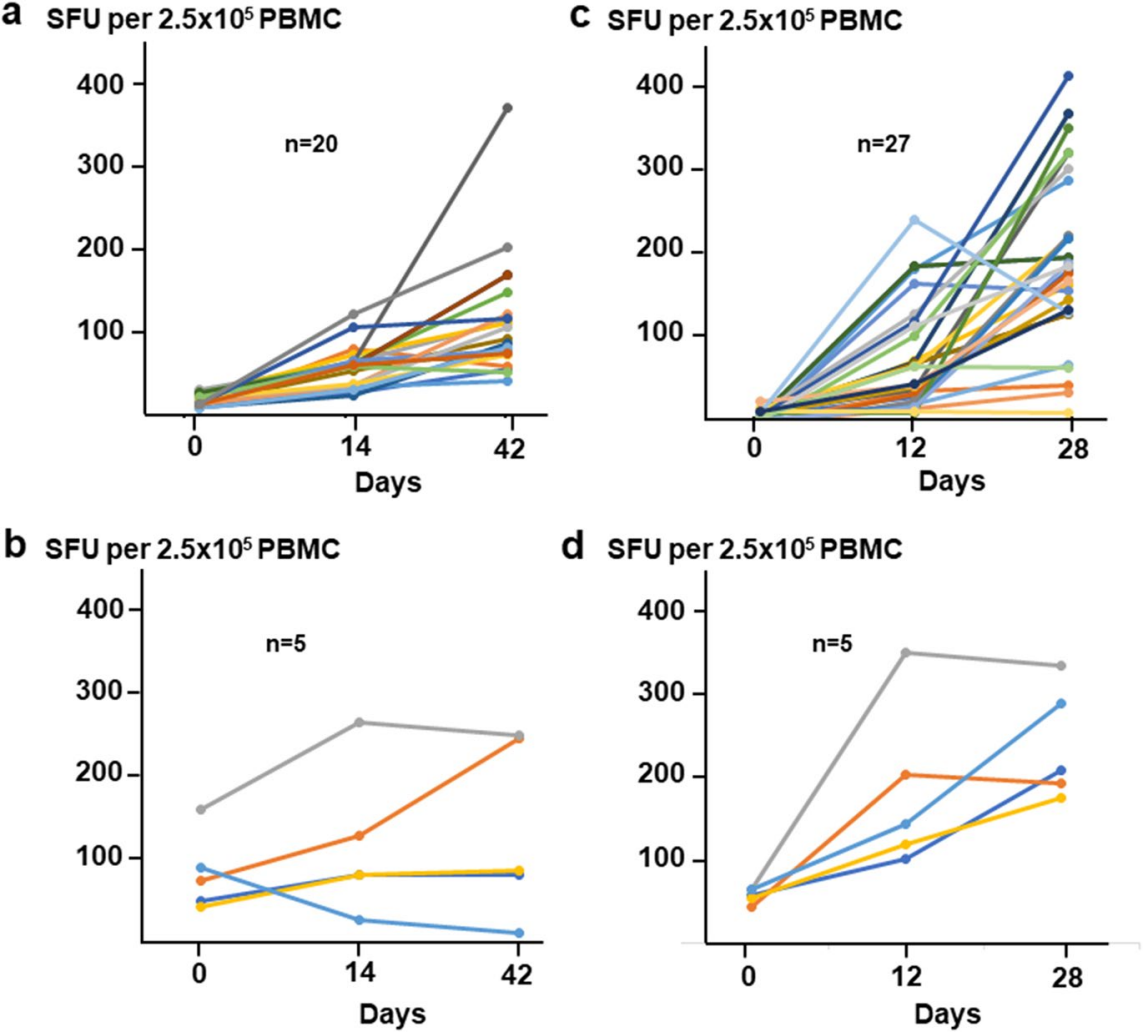
Magnitude of BBIBP-CorV and BNT162b2-induced T cell responses. The cumulative SFU (anti-S1 + S2 + N + M + E) responses for 25 individuals that received the BBIBP-CorV vaccine (**a** and **b**) and for the 32 participants that received the BNT162b2 vaccine (**c** and **d**) were measured by the T-SPOT® Discovery SARS-CoV-2 ELISpot assay. Different panels demonstrate T cell responses in individuals with (**b** and **d**) or without (**a** and **c**) preexisting T cell immunity (≥ 40 or ˂ 40 SFU/2.5 × 105 PBMC, respectively) at day 0. Connected lines indicate repeated measurements from the same subjects

Anti-SARS-CoV-2 T cells responsive to the spike (S), nucleocapsid (N), membrane (M), and envelope (E) peptide pools were measured using the IFNγ ELISpot assay. Overall, the magnitude of cumulative T cell response was higher in the BNT162b2 cohort (median 209.4 SFU, group 1, at day 28) than in the BBIBP-CorV vaccinated participants (median 103.9 SFU, group 1, at day 42, *p* < 0.01) (Fig. 5a, c). Only one BNT162b2-injected, naive individual had no response to any of the peptide pools. This 56-year-old male participant was also seronegative after boosting, i.e., clearly non-responder to BNT162b2. In another volunteer from the second group of the BBIBP-CorV cohort, a marked reduction in the anti-SARS-CoV-2 T cell response was seen under vaccination (Fig. 5b), despite he was asymptomatic and PCR negative at day 0. Notably, cumulative SFU numbers in SARS-CoV-2 experienced groups (Fig. 5b, d) were slightly but not always significantly higher than in virus-naive individuals.

**Fig. 6:**
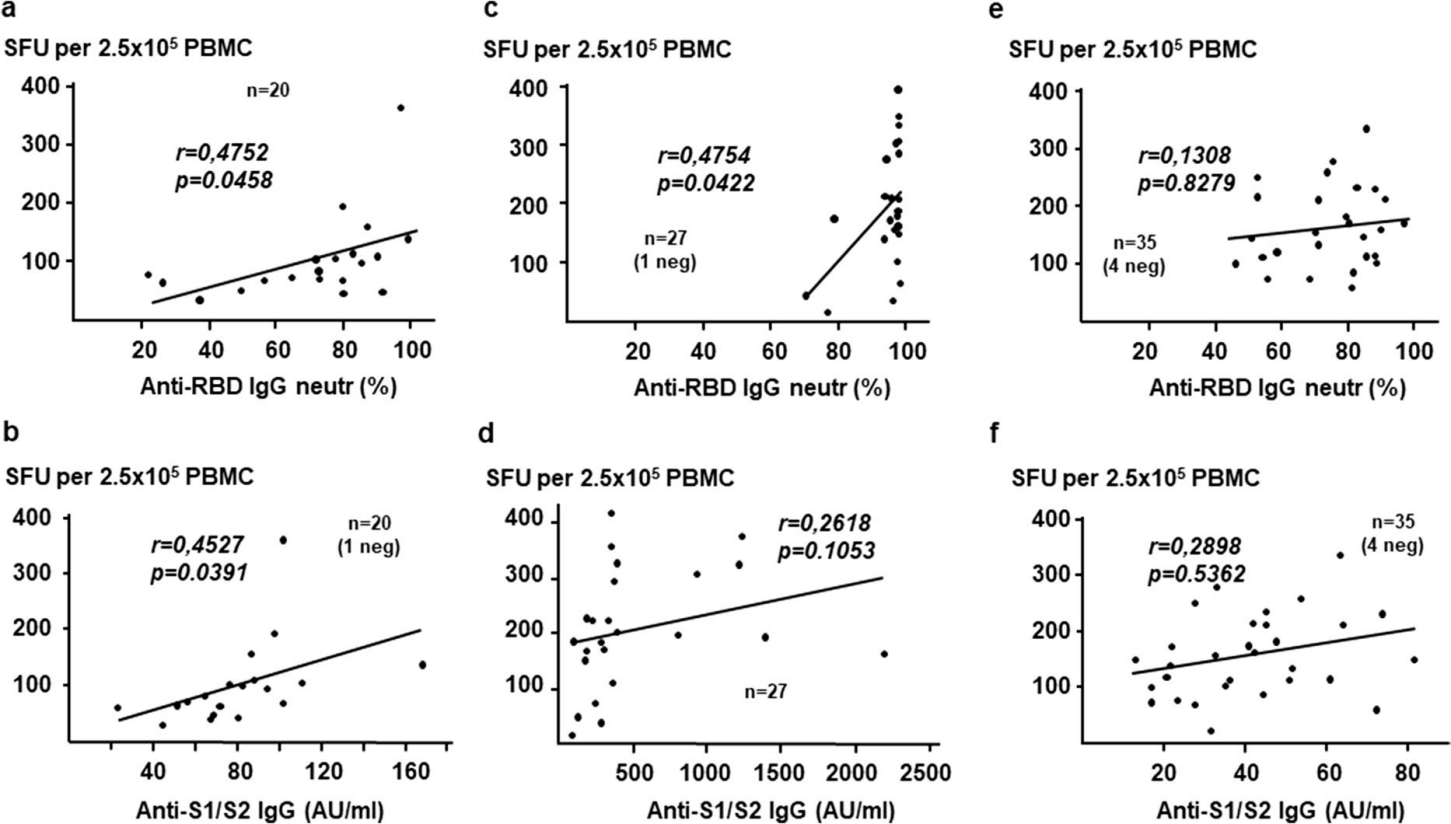
Correlation of antibody and T cell responses induced by different SARS-CoV-2 vaccines. Correlations between anti-RBD IgG (**a**, **c** and **e**) and anti-S1/S2 IgG (**b**, **d** and **f**) levels and the cumulative SFU responses after the second vaccine dose in 20 virus-naive (group 1) individuals that received BBIBP-CorV (**a** and** b**), in 27 virus-naive (group 1) individuals that received BNT162b2 (**c** and **d**), and in 35 individuals that recovered from COVID-19 disease (**e** and **f**), as assessed by Pearson correlation. R, correlation coefficient. Neg, seronegative

Next, we performed Pearson correlation coefficient analysis between T cell and antibody responses in both cohorts. As shown in Fig. 6a and c, SFU numbers strongly correlate with the anti-RBD IgG levels in virus-naive groups. Similar results were found in anti-S1/S2 IgG in BBIBP-CorV immunized virus-naive participants (Fig. 6b). However, no significant positive correlation was observed between IFNγ-secreting T cells and anti-S1/S2 antibody levels in BNT162b2 vaccinated virus-naive individuals (Fig. 6d). Interestingly, we did not see either any significant correlation between the magnitude of SARS-CoV-2-specific T cell and IgG responses in convalescent patients (Fig. 6e, f).

Finally, correlation between spike protein-specific serum IgA levels and SFU numbers was found neither in the BBIBP-CorV nor in the BNT162b2 cohorts (first groups, Supplementary Fig. 2a, b). It should be emphasized that the specificity of anti-SARS-CoV-2 T cells was markedly different in the two, i.e., in the BBIBP-CorV or BNT162b2 vaccinated cohorts. The second dose of BBIBP-CorV vaccine elicited a median SFU of 33.6 for S1 peptide pool, 20.6 for S2 pool, 35.9 for the nucleocapsid pool, and 15.5 for the membrane pool, compared to 114.9, 74.2, 4.1, and 4.5 in BNT162b2 immunized naive (first groups) participants, respectively. Thus, BNT162b2 was unable to elicit T cell responses against N and M proteins, which is not encoded in the mRNA vaccine. Notably, some, but not significant, anti-N (median SFU = 17.2) and anti-M (median SFU = 11.6) T cell responses could be observed in mRNA-vaccinated, virus-experienced individuals (BNT162b2 cohort, group 2). Indicators of previous SARS-CoV-2 infection were also seen in the BBIBP-CorV cohort. In this case, the anti-N and anti-M SFU numbers were significantly higher in virus-experienced participants (group 2), compared to naive volunteers (group 1) (60.8 and 28.5 versus 35.9 and 15.5, respectively) (Table 2).

**Table 2:** Specificity of IFN-γ^+^ anti-SARS-CoV-2 T cells after boosting

	Peptide pools					
	Nil	S1	S2	N	M	E
BBIBP-CorV						
Group 1 (*n* = 20)	1.2* (0–5)	33.6 (10–125)	20.6 (5–47)	35.9 (6–127)	15.5 (0–61)	3.6 (0–16)
Group 2 (*n* = 5)	0.0	41.0 (18–78)	35.8 (15–75)	60.8 (26–101)	28.5 (8–55)	7.0 (0–19)
BNT162b2						
Group 1 (*n* = 27)	2.0 (0–10)	114.9 (4–261)	74.2 (5–171)	4.1 (0–9)	4.5 (0–18)	15.3 (0–59)
Group 2 (*n* = 5)	0.6 (0–2)	114.6 (79–136)	83.8 (47–137)	17.2 (2–42)	11.6 (0–25)	16.0 (1–58)
Convalescent individuals (*n* = 35)	3.9 (0–18)	35.2 (3–81)	29.2 (1–78)	33.8 (0–85)	34.2 (3–84)	9.8 (0–62)

^*^Spot-forming units (median; range)

Overall, the magnitude and specificity of T cell response to SARS-CoV-2 structural proteins (S, N, and M) were similar in BBIBP-CorV vaccinated healthy volunteers and in convalescent patients. In contrast, the mRNA-based BNT162b2 vaccine was able to induce much stronger T cell response to the spike protein (S1 and S2 peptide pools) but unable to elicit any anti-nucleocapsid or anti-membrane protein-specific T cell response.

## Discussion

In this observational study, we compared the antibody and T cell responses to the BBIBP-CorV (inactivated virus technology based) and the BNT162b2 (mRNA technology based) vaccines in healthy adult volunteers. To our best knowledge, our work represents the first comparative analysis of adaptive immunity to these vaccines.

Blood samples were obtained from all participants prior vaccination and following their first and second doses. Much higher anti-SARS-CoV-2 antibody levels were measured among virus-naive individuals vaccinated with two doses of BNT162b2 compared to BBIBP-CorV, but all individuals were still seroconverted in the BBIBP-CorV cohort as well. For example, anti-S1/S2 IgG levels were ~ 6–sevenfold lower in BBIBP-CorV vaccinated naive individuals compared with the BNT162b2 naive group after boosting. The cumulative number of IFNγ-secreting, virus-specific T cells, however, was less different in mRNA and inactivated virus vaccinated participants. In addition, BNT162b2 was able to induce anti-spike T lymphocyte responses only, while in the BBIBP-CorV cohort, T cell responses against the nucleocapsid and membrane proteins, which are not encoded in the mRNA vaccine, were also observed. Thus, BBIBP-CorV could provoke a T cell response in naive individuals that is comparable to that seen in convalescent patients.

Although the exact immunological correlates of protection against COVID-19 remain unknown, it is clear that both virus-specific antibodies and T lymphocytes are present in patients who recovered from the illness [10]. It was shown that suboptimal T cell response contributes to COVID-19 severity in many patients [11]. Sometimes robust T cell response was observed even in seronegative individuals with asymptomatic or mild disease [12]. Moreover, it has been demonstrated that new variant viruses (VOCs, variants of concern) which are able to partially escape antibody response induced by the original (Wuhan-1) virus do not significantly disrupt the total SARS-CoV-2 T cell reactivity [13, 14] and the formation of long-lasting T cell memory [15]. The mRNA vaccine, however, induces T cell responses that narrowly target the spike protein most prone to mutations, whereas the inactivated virus vaccine creates much broader responses against epitopes of spike, nucleocapsid, and membrane proteins. In other words, BBIBP-CorV vaccine targeting multiple epitopes, compared with BNT162b2, could further mitigate the possibility of immune escape by new mutations. Additionally, the CD8^+^ effector T cells [15] and the long-lived, self-renewing CD8^+^ memory cells are mainly anti-N-specific in both infected and convalescent patients [16, 17]. Notably, the anti-N IgG antibodies, or at least part of them, have virus-neutralizing activity [18]. Therefore, the immune responses targeting the nucleocapsid determinants could be important to induce protection against different coronavirus infections, including VOCs. Of course, the relative contributions of anti-S versus anti-N responses in the induction and maintenance of effective anti-SARS-CoV-2 immunity after vaccine challenge remain to be determined.

Consistent with several previous publications [19], in antibody and T cell responses, dramatic variabilities were observed between study participants across all assays. It is likely a consequence of the genetic variability among individuals, including different allelic versions of the HLA genes and other, more or less identified genetic loci [20]. The individual differences were higher in the BNT162b2 cohort compared to BBIBP-CorV, which could also be explained by the lower number of immunodominant epitopes in the spike-only (mRNA) vaccine compared with the inactivated SARS-CoV-2 virus. Based on these and other published data [17, 21–23], we believe that new COVID-19 vaccines must contain immunodominant determinants from more than one protein of the SARS-CoV-2 virus. Alternatively, heterologous prime-boost (for example, BBIBP-CorV and BNT162b2) vaccination may be used to increase vaccine effectiveness.

In summary, two doses of the BBIBP-CorV inactivated virus vaccine are able to induce modest anti-SARS-CoV-2 antibody and strong, comprehensive T cell responses in all healthy adult individuals. However, the duration of vaccine elicited adaptive immune responses need further investigation. This question is being addressed by an ongoing study at our institute.

The main limitations of our work include a small sample size, a short follow-up period, and a lack of representation of children, adolescents, and older adults.

## Supplementary Information

Below is the link to the electronic supplementary material.

Supplementary file1 (PDF 192 KB)
