# Supplementary material for: Comparison of antibody and T cell responses elicited by BBIBP-CorV (Sinopharm) and BNT162b2 (Pfizer-BioNTech) vaccines against SARS-CoV-2 in healthy adult humans
Source: GeroScience. 2021 Oct 11;43(5):2321–31. doi: 10.1007/s11357-021-00471-6 (PMC8503874; doi:10.1007/s11357-021-00471-6)
Supplement: Supplementary file 1 — Supplementary file1 (PDF 192 KB) [file 11357_2021_471_MOESM1_ESM.pdf]

Comparison of antibody and T cell responses elicited by BBIBP-CorV (Sinopharm) and BNT162b2 (Pfizer-BioNTech) vaccines against SARS-CoV-2 in healthy adult humans

István Vályi-Nagy[1], Zsolt Matula[2], Márton Gönczi[3], Szabolcs Tasnády[3], Gabriella Bekő[3], Marienn Réti[1], Éva Ajzner[1] and Ferenc Uher[2]

[1]Department of Hematology and Stem Cell Transplantation, Central Hospital of Southern Pest, National Institute of Hematology and Infectious Diseases, Budapest, 1097 Hungary
[2]Laboratory for Experimental Cell Therapy, Central Hospital of Southern Pest, National Institute of Hematology and Infectious Diseases, Budapest, 1097 Hungary
[3]Central Laboratory of Central Hospital of Southern Pest, National Institute of Hematology and Infectious Diseases, Budapest, 1097 Hungary

Corresponding author:
Ferenc Uher, PhD,
Central Hospital of Southern Pest – National Institute of Hematology and Infectious Diseases,
Nagyvarad ter 1.
Budapest, Hungary, H-1097;
Phone number: +36 06 666 3911
Fax number: +36 06-1-216-1493
E-mail: uher.ferenc@gmail.com

Supplementary Table 1. **Anti-NP IgG response after the second vaccine dose**

	**BBIBP-CorV**		**BNT162b2**	
	**Group 1**	**Group 2**	**Group 1**	**Group 2**
**No of individuals per positive individuals**	20/7	5/4	27/0	5/1

Supplementary Fig. 1. **T lymphocyte landscape in participants vaccinated with BBIBP-CorV.** Box plots of major T cell subsets from PBMCs of individuals immunized with BBIBP-CorV. Different panels demonstrate the absolute numbers of $CD3^{+}$ T (a and b), $CD3^{+}CD4^{+}$ T (c and d), and $CD3^{+}CD8^{+}$ T cells (e and f) in individuals with (b, d and f) or without (a, c and e) preexisting T cell immunity at day 0. Box plots display the median values with the interquartile range (lower and upper hinge) and ±1.5 fold the interquartile range from the first and third quartile (lower and upper whiskers). P-values are determined by the Wilcoxon signed rank test and $p < 0.05$ was considered to be statistically significant (*$p<0.05$; **$p<0.01$; ***$p<0.001$).

Supplementary Fig. 2. **Correlation of serum anti-S IgA and T cell responses induced by different SARS-CoV-2 vaccines.** Correlations between anti-S IgA and the cumulative SFU responses after the second vaccine dose in 20 virus-naive (group 1) individuals that received BBIBP-CorV (a) and 27 naive (group 1) individuals that received BNT162b2 (b), as assessed by Pearson correlation. R, correlation coefficient. Neg, seronegative.

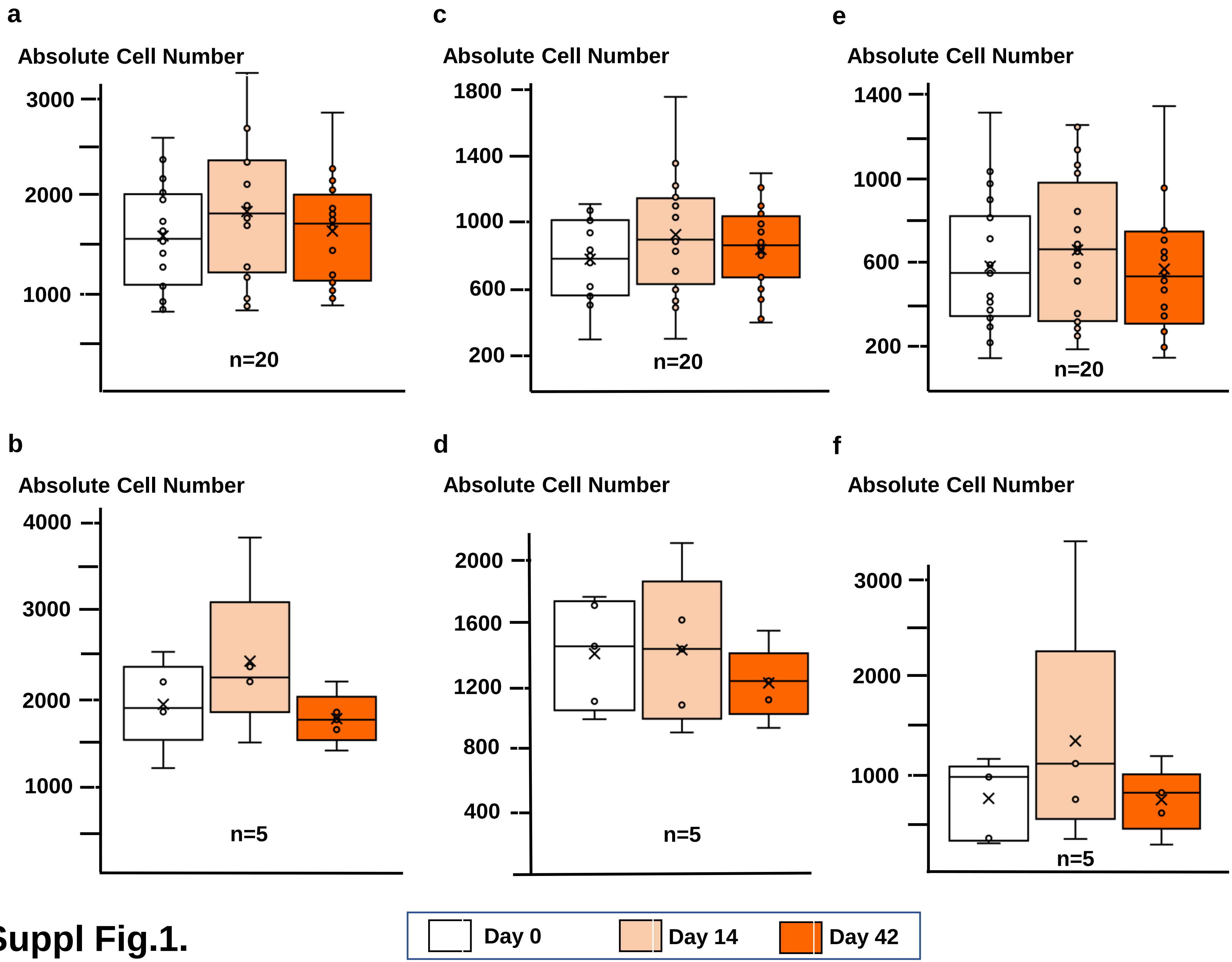
a
Absolute Cell Number
3000
2000
1000
n=20
c
Absolute Cell Number
1800
1400
1000
600
200
n=20
e
Absolute Cell Number
1400
1000
600
200
n=20
b
Absolute Cell Number
4000
3000
2000
1000
n=5
d
Absolute Cell Number
2000
1600
1200
800
400
n=5
f
Absolute Cell Number
3000
2000
1000
n=5
Day 0
Day 14
Day 42

Suppl Fig.1.

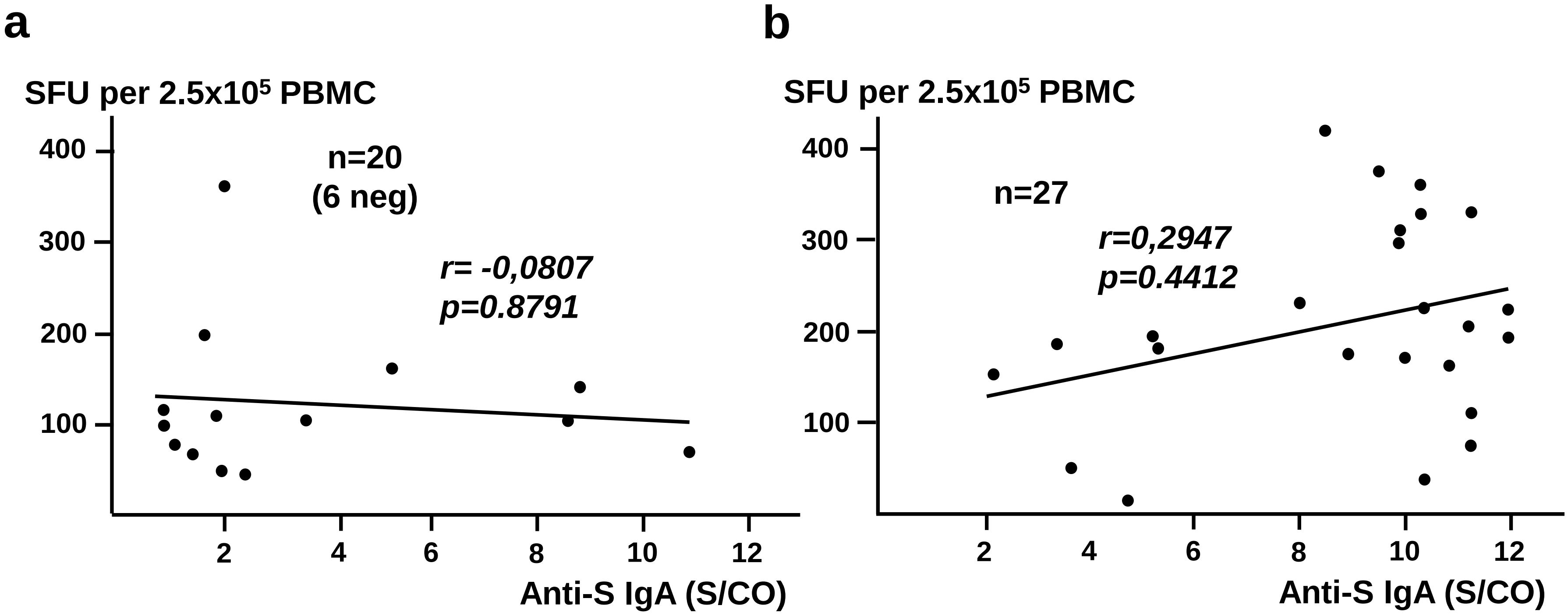
a
SFU per 2.5x10⁵ PBMC
400
300
200
100
n=20
(6 neg)
r= -0,0807
p=0.8791
2
4
6
8
10
12
Anti-S IgA (S/CO)
b
SFU per 2.5x10⁵ PBMC
400
300
200
100
n=27
r=0,2947
p=0.4412
2
4
6
8
10
12
Anti-S IgA (S/CO)

Suppl. Fig.2.
